# An Outbreak of Food Poisoning by Salmonella Typhimurium in a District of Lower Assam in North-East India

**DOI:** 10.7759/cureus.117702

**Published:** 2026-10-09

**Authors:** Suranjana C Hazarika, Mehzabin H Hazarika, Cornelioush Basumatary, Lakshyasri Baishya, Himanto Hazarika, Ajanta Sharma, Purabi Deka, Madhuchhanda Das, Samaresh Das, Thandavarayan Ramamurthy

**Affiliations:** 1 Microbiology, Gauhati Medical College and Hospital, Guwahati, IND; 2 Community Medicine, Pragjyotishpur Medical College and Hospital, Guwahati, IND; 3 Allied Health Sciences, Srimanta Sankaradeva University of Health Sciences, Guwahati, IND; 4 Ophthalmology, Nalbari Medical College and Hospital, Nalbari, IND; 5 Microbiology, Lakhimpur Medical College and Hospital, Lakhimpur, IND; 6 Development Research, Indian Council of Medical Research, New Delhi, IND; 7 Health Informatics and Allied Technologies, Centre for Development of Advanced Computing, Kolkata, IND; 8 Bacteriology, National Institute of Cholera and Enteric Diseases, Kolkata, IND

**Keywords:** epidemic curve, food poisoning, outbreak investigation, public health, salmonella typhimurium

## Abstract

Introduction

Foodborne infection is a neglected health problem. Although large numbers of focal outbreaks are reported every year, implementation of strong food safety policies and guidelines is still awaited due to the paucity of data. Here, we report an outbreak of food poisoning from a district of Lower Assam where 65 people (attack rate 65%) reported sick in a span of a few hours in a particular locality.

Materials and methods

An outbreak investigation was conducted as part of a task force project of the Indian Council of Medical Research* (*ICMR) on foodborne pathogen surveillance in North-East India, in collaboration with the Integrated Disease Surveillance Programme (IDSP) team. A combined retrospective-prospective study was conducted. Data on patient demographics, time of onset of symptoms, presenting clinical features, and history of food items consumed were collected. Stool samples from cases were also collected for examination as per standard operating procedures (SOP).

Results

Most cases complained of abdominal cramps (n = 60, 92%), fever (n = 59, 90%), and diarrhoea (n = 55, 84%) about 48-72 hours after consumption of the food. 'Prasad' (an offering comprising chickpea or bengal gram, moong or green gram, fruits, etc., and served raw) was thought to be the implicating food since it was consumed by 100% of cases. *Salmonella *Typhimurium was isolated from three of the seven stool samples collected, which showed resistance to cotrimoxazole, ampicillin, and ciprofloxacin.

Conclusion

The outbreak was most probably associated with the consumption of raw 'Prasad' contaminated with *S.* Typhimurium identified in stool samples and showing multidrug resistance. Strengthening food hygiene practices and timely foodborne disease surveillance are essential to prevent similar outbreaks in the future. Recommendations were made for proper hygiene during preparation, handling, and storage of food.

## Introduction

A foodborne outbreak is a public health emergency, posing a huge burden on the health and economy of the country. It is important to investigate an outbreak promptly to curtail its spread, identify the causative agent and source of infection and reduce associated mortality and morbidity. Although the Government of India has implemented several national programmes such as the Swachh Bharat Mission and the National Rural Drinking Water Programme (NRDWP) to improve sanitation and hygiene and reduce disease burden, there remains a critical need to enhance public awareness and practices related to food safety and hygiene [1-3].

Food poisoning is an acute illness that occurs after ingesting food or water contaminated with micro-organisms. It may be either infectious or non-infectious in nature. Infectious food poisoning results from the consumption of food or water contaminated with bacteria, fungi, viruses, parasites or the toxins produced by these microorganisms. It is also commonly referred to as a foodborne disease. The most common symptoms include nausea, vomiting, abdominal cramps, diarrhoea, headache and malaise. In some cases, additional symptoms, such as fever and abdominal pain, may also be present [4]. A food poisoning outbreak is defined as the occurrence of two or more cases of a similar foodborne illness resulting from the consumption of a common source of food [5].

Microorganisms most frequently responsible for food poisoning include *Staphylococcus aureus, Salmonella, Clostridium perfringens, Campylobacter* and *Norovirus* [4,5]. Most hospitalisations related to food poisoning are attributed to *Salmonella*, *Norovirus*, *Campylobacter*, *Toxoplasma gondii* and* Escherichia coli* [4]. Fatal outcomes are most commonly associated with infections caused by *Salmonella*, *Toxoplasma gondii*, *Listeria monocytogenes*, *Norovirus* and *Campylobacter* [4].

The duration of food poisoning varies depending on its cause, but most cases typically begin within a few hours of consuming contaminated food or drink and may last for several days [4]. Foods commonly implicated in food poisoning include eggs, poultry and meat, unpasteurised milk and dairy products, raw or unwashed fruits and vegetables, as well as nuts and spices [4,6]. Factors contributing to foodborne outbreaks include consumption of undercooked or improperly thawed meat or poultry, cross-contamination from infected food handlers and the presence of pests such as flies, cockroaches and rats in the food environment, which can act as vectors of the disease [7-9]. An estimated 866 million - almost one in nine individuals in the world - fall ill after eating contaminated food, and 1.52 million die every year. Children under five years of age experience 29% of the health burden due to unsafe food, with 143,000 deaths in 2021 [10]. Although the highest number of cases and deaths of food poisoning occur in developing countries [7], there is limited evidence regarding the verification of the occurrence of outbreaks, detailed case descriptions and predictors of food poisoning outbreaks in the study area.

Non-typhoidal salmonellosis is one of the leading foodborne illnesses and contributes substantially to morbidity and mortality in both developed and developing regions. For example, in the United States, non-typhoidal salmonellosis has been estimated to account for 11% (ranking second) of foodborne illnesses, 35% (ranking first) of foodborne-associated hospitalisations and 28% (ranking first) of foodborne-associated deaths [11]. *Salmonella *Typhimurium and *Salmonella *Enteritidis are the most prevalent *Salmonella* serotypes causing human gastroenteritis worldwide [12]. An outbreak of gastroenteritis caused by non-typhoidal *Salmonella* (*Salmonella *Weltevreden) was reported from a tea garden in Assam, North-East India, which was due to contaminated water [13]. Another study from North Dumdum, West Bengal, in India reported non-typhoidal *Salmonella *(*Salmonella enterica* serovar Weltevreden) as the etiological agent of a foodborne gastroenteritis outbreak after an Iftar feast [14]. A study by Kumar et al. that reported the disease burden of non-typhoidal Salmonellosis from across India reported the predominant organism to be *S. *Typhimurium(30.98%) followed by *S. *Lindenberg(25.05%), *S. *Enteritidis (10.39%), *S. *Weltevreden(8.16%), etc [15]. The ability of *Salmonella* to withstand environmental stresses makes it difficult to eradicate this pathogen from the food chain [16]. Large-scale studies based on foodborne outbreak data indicate that the most common contributing factors associated with foodborne salmonellosis include cross-contamination, inadequate cooling or refrigeration, insufficient heat treatment and contamination by food handlers [17,18]. Even though *S. *Typhimuriumtypically causes self-limiting gastroenteritis [19], it can also cause other infections such as bacteremia, focal extra-intestinal infections, meningitis and osteomyelitis, particularly among children [20,21]. Additionally, the increasing prevalence and spread of antimicrobial resistance within *Salmonella* species [22], accompanied by the lack of new antimicrobial agents, presents a global concern.

In India, data on food- and waterborne infections and outbreaks are scarce. Most outbreaks are either not reported or not investigated properly and published. In view of such a data gap, the Indian Council of Medical Research has conducted a surveillance study of food and waterborne pathogens in the North-East region of India. Gauhati Medical College and Hospital is one of the centres of the study, and outbreak investigation is one of the key components of the study. In this study, we report an acute diarrhoeal disease outbreak that occurred in Ekhrabil village of Nagrijuli under Tamulpur sub-division, Baksa. To the best of our knowledge, this is the first complete outbreak investigation in this region, which could significantly contribute to the understanding of a neglected public health issue.

The investigation aimed to confirm the food-poisoning outbreak, describe cases by person, place and time, and identify associated factors in Ekhrabil village of Nagrijuli. It also sought to provide recommendations to control the outbreak and prevent similar incidents in the future.

## Materials and methods

Study context

On 15 April, 2023, the Gauhati Medical College team received information from the State Surveillance Officer of the Integrated Disease Surveillance Programme (IDSP) that a food poisoning outbreak had occurred at Ekhrabil village of Nagrijuli under Tamulpur Sub-division, Baksa. Several patients had been admitted to the Nagrijuli Model Hospital with complaints of fever, abdominal cramps, diarrhoea, headache, vomiting, etc. The project staff immediately visited the hospital along with the rapid response team and the team from IDSP for verification of the outbreak. The team interacted with the hospital staff and patients about the incident. 

Study setting

An outbreak of acute gastroenteritis occurred at Ekrabil village, Nagrijuli, Baksa district of Assam on 15 April, 2023. About 100 people attended a religious ceremony (Ganesh Puja) at a teashop in Nagrijuli on 13 April, 2023, where lunch was also served after the Puja ceremony. A total of 65 people were affected. Most affected people were admitted to the Nagrijuli Model Hospital for treatment. The team also collected the details of food items served during the ceremony such as 'Prasad' (an offering comprising chickpea or bengal gram, moong or green gram, fruits, etc., and served raw) and Khichri (a cooked dish made of rice, lentils, vegetables and ghee), and water. The outbreak continued till 17 April 2023.

Study design

A combined retrospective-prospective approach was adopted to investigate the outbreak by the Gauhati Medical College and Hospital team in collaboration with the IDSP unit of the Health Department, Baksa. All people (n=100) who attended the Puja ceremony were included in this outbreak investigation. This included 65 affected people (test group) and 35 unaffected people (control group). The data that were collected retrospectively included information regarding the food consumed in the Puja ceremony, the number of people who attended the function, as well as the number of people who were affected, date and time of onset of symptoms, etc. Prospectively, stool samples were collected from the affected patients from whom it was possible, and processed, as well as water samples from the place where the implicated food was prepared.

Case definition

A probable case of food poisoning was defined as a previously healthy individual who suddenly developed any two of the following symptoms after taking food at the Puja ceremony: abdominal cramps, fever, diarrhoea, headache, nausea or vomiting. Acute watery diarrhoea is defined as the passage of three or more loose or watery (non-bloody) stools within a 24-hour period [23].

Data collection

The team collected the data from people who had attended the Puja ceremony through a structured questionnaire (epidemiological case sheet) regarding demographic particulars of individuals (Appendices). A line listing of all these individuals was prepared. The project team gathered information from all affected individuals, including those admitted to the Nagrijuli Model Hospital, focusing on the time and date of symptom onset, clinical presentation and history of food consumption during the ceremony. All 65 affected individuals were interviewed. We used a standardised case report form to collect both demographic and clinical information. Data recorded included age, sex, signs and symptoms, date of illness onset, date of hospital admission, history of food intake, date of discharge, treatment received and outcomes.

The team also collected stool samples from patients for identification of causative enteric pathogens by laboratory investigation. Two water samples, one from the tube-well and the other from the SINTEX reservoir, were also collected for analysis from the place where the ceremony was held. No food sample was collected as no suspected food item was left by the time the symptoms appeared (after 48 hours) and when the news of the outbreak reached IDSP. The collected stool and water samples were tested for possible foodborne pathogens in the Microbiology Department of Gauhati Medical College and Hospital, Guwahati. A detailed inspection of the teashop was conducted to assess the hygiene and sanitary conditions, food preparation and storage practices and the presence of rodent infestation.

Sample collection and transportation

All samples were collected and transported as per the standard operating procedures (SOP) [24]. Cary-Blair medium was used for sample collection and transportation. The cold chain was maintained during sample transportation.

Laboratory investigation

Microscopy, culture and antibiotic sensitivity testing were done following the standard protocol. Seven stool samples were collected from patients for culture and sensitivity tests (the sample size was determined as per the IDSP guidelines, which state that 'in large outbreaks, specimens must be collected from at least 10% of cases') [25]. Stool samples were processed for possible pathogens of food poisoning following the SOP for processing stool samples. We inoculated the collected stool samples in Selenite F broth for enrichment along with other enrichment media. After incubation at 37°C for 18-24 hours, subcultures were made from Selenite F broth on MacConkey agar and xylose lysine deoxycholate (XLD) agar. In three of the samples, colourless or pale, small non-lactose-fermenting colonies were seen on MacConkey agar, while colonies with black centres were seen in the XLD medium due to the production of hydrogen sulphide (H_2_S), commonly seen in the case of *Salmonella *species. The colonies were sub-cultured on nutrient agar plates and incubated at 37°C for 18-24 hours to perform various biochemical tests, including Simmons citrate agar, Christensen's urea agar, indole production, urease, triple sugar iron (TSI) agar, lysine iron agar (LIA) and motility, indole, ornithine (MIO) tests. Further identification was carried out using a slide agglutination test with *Salmonella* O and H polyvalent antisera.

*Salmonella* are Gram-negative, aerobic and facultative anaerobic motile bacilli. They are members of the Enterobacteriaceae family. *Salmonella enterica* and *Salmonella bongori *are the two main species of the genus *Salmonella*. These two species are further divided into more than 2,500 serovars or serotypes. Most pathogenic species of *Salmonella* causing illness in humans belong to the *Salmonella enterica* species [26]. Within *Salmonella enterica* subspecies *enterica*, the subspecies designation (subspecies enterica) is typically omitted, as only serovars within this subspecies are formally named [27]. Accordingly, in this outbreak study, serotypes such as *Salmonella enterica *subspecies enterica serovar Typhimurium have been referred to as *Salmonella* Typhimurium or simply *S. *Typhimurium.

Antibiotic sensitivity tests were done using the Kirby-Bauer disk diffusion method on Mueller-Hinton agar plates. The plates were incubated at 37°C for 16-18 h. After incubation, the zones of inhibition obtained around each antibiotic disk were measured and recorded. Interpretation of the zone size was done according to the SOP [24]. Antibiotic disks used were ampicillin (10 µg), chloramphenicol (30 µg), azithromycin (15 µg), cefotaxime (30 µg), cefepime (30 µg), ceftriaxone (30 µg), ciprofloxacin (5 µg), gentamicin (10 µg), imipenem (10 µg), meropenem (10 µg), nalidixic acid (30 µg), trimethoprim-sulphamethoxazole (1.25/23.75 µg) and tetracycline (30 µg).

Confirmation of diagnosis

The isolated strains were confirmed at the ICMR - National Institute for Research in Bacterial Infections (NIRBI) located in Kolkata, which is also the External Quality Assessment Scheme (EQAS) centre in the Task Force study. 

Data analysis

For analysis, the data were transferred to a Microsoft Excel spreadsheet (Microsoft Corp., Redmond, Washington). The analysed data were presented by text, tables and graphs. Collected data were entered into Epi Info version 7 (developed by the Centers for Disease Control and Prevention (CDC)) for the creation of epidemiological curves (epi curve) and analysis of epidemiological data. The association of food consumption between the control group (35) of all unaffected people who had attended the religious ceremony and the test group (65) was statistically calculated using the chi-square test.

Ethical consideration

The project team conducted the outbreak investigation after obtaining permission and clearance from the Joint Director of Health Services, Baksa district. Informed consent was taken from the participants before conducting the interview. For participants under the age of 18 years, informed written consent was also obtained from a parent or guardian on behalf of the participant. The participants’ right to refuse participation or to withdraw at any time during the interview was respected. The confidentiality of all information obtained from participants was strictly maintained. The study was conducted in accordance with the Declaration of Helsinki and ethical clearance was obtained from the institutional ethics clearance committee of Gauhati Medical College and Hospital (ref. no. MC/190/2007/Pt-11/Oct-2019/19, dated 06.11.2019).

## Results

Out of a total of 100 people who attended the Puja ceremony, 65 people were affected. Thus, the attack rate in this outbreak was 65%. Symptoms first appeared on 15 April, with peak incidence on 16 April.

The most common symptom was abdominal cramps followed by fever and diarrhoea (Table 1).

**Table 1:** Number of symptoms (out of a total of 65 patients)

Value	Number	Percentage (%)
Abdominal cramps	60	92.31
Fever	59	90.77
Diarrhoea	55	84.62
Body ache	47	72.31
Headache	29	44.62
Vomiting	20	30.77
Nausea	16	24.62
Malaise	3	4.62

Descriptive epidemiology with person, place and time

Distribution by Gender

There was not much difference in the frequency between male and female patients; both were almost equally affected, as shown in Table 2.

**Table 2:** Frequency of gender distribution in test group and control group

Test group (n = 65)		Control group (n = 35)	
Male	35 (53.85%)	Male	19 (54.29%)
Female	30 (46.15%)	Female	16 (45.71%)

Distribution by Age

The age of affected individuals ranged from 2 to 62 years, with a mean age of approximately 24 years (Table 3). 

**Table 3:** Age distribution of test and control groups

Age group	Test group (n = 65)	Control group (n = 35)
0 to <10 (years)	14 (21.54%)	6 (17.14%)
10 to <20 (years)	19 (29.23%)	11 (31.42%)
20 to < 30 (years)	10 (15.38%)	7 (20%)
30 to < 40 (years)	8 (12.31%)	4 (11.42%)
40 to < 50 (years)	9 (13.85%)	5 (14.28%)
50 to < 60 (years)	3 (4.62%)	2 (5.71%)
≥ 60 (years)	2 (3.08%)	0
Total	65 (100%)	35 (100%)

Distribution by Place

The outbreak of gastroenteritis occurred in the village of Ekrabil under Tamulpur subdivision, Baksa district of Assam, India, following consumption of food at a religious or 'Puja' ceremony at a teashop present in the locality.

Distribution by Time

Symptoms first appeared on 15 April, with a peak incidence on 16 April (more than 50 cases), as shown in Figure 1. Most patients were admitted to the Nagrijuli Model Hospital.

**Figure 1:**
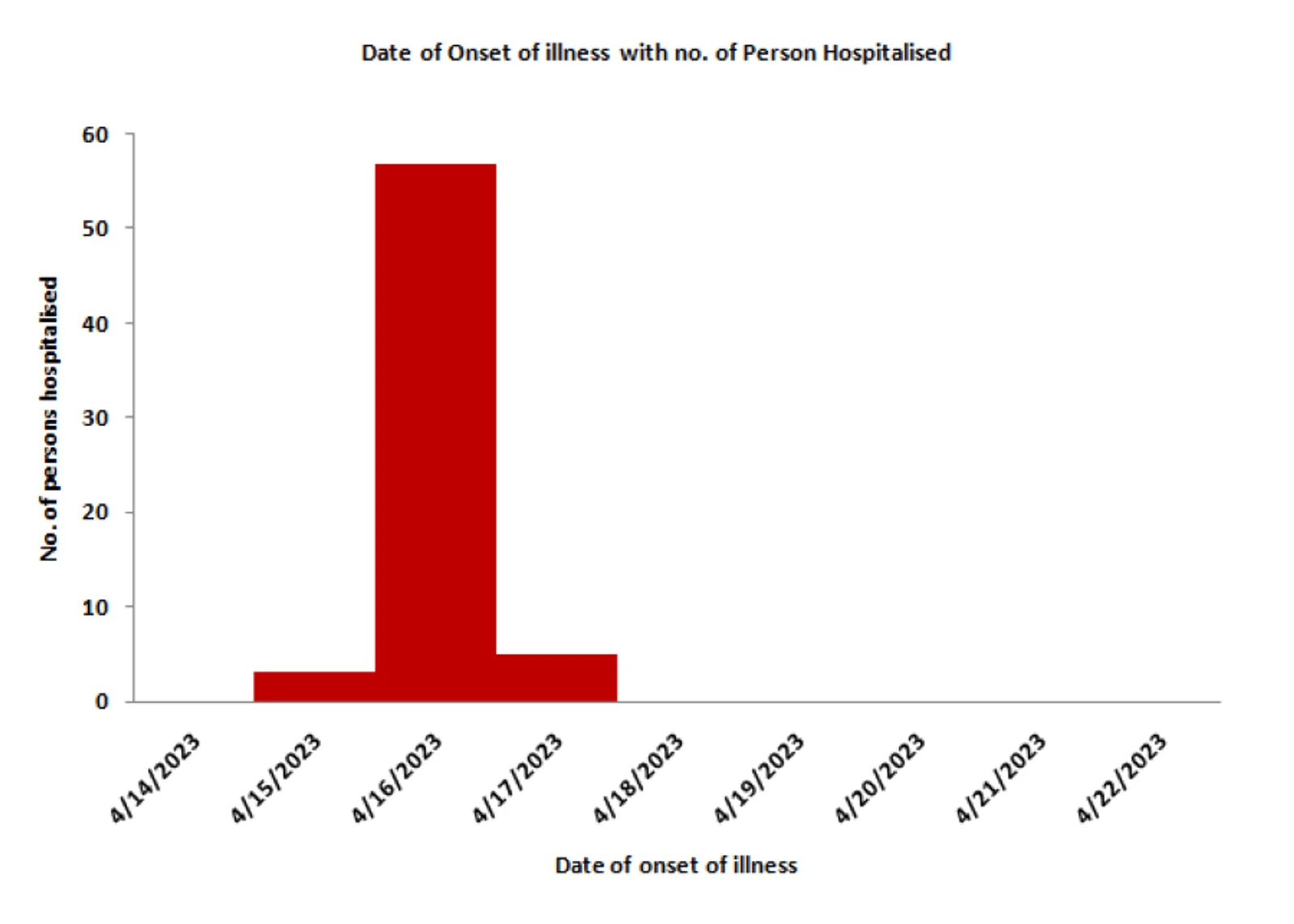
Distribution of cases according to the date of onset The figure was created using Epi Info^TM^ 7, developed by the Centers for Disease Control and Prevention (CDC).

The epidemic curve showed a point-source outbreak from a common source. There was a peak in incidence on 16 April, around 4:00-8:00 a.m. and again around 17:00-20:00 p.m. (Figure 2). There were no outliers in this outbreak. All cases occurred between 15 and 16 April, and the outbreak subsided on 17 April.

**Figure 2:**
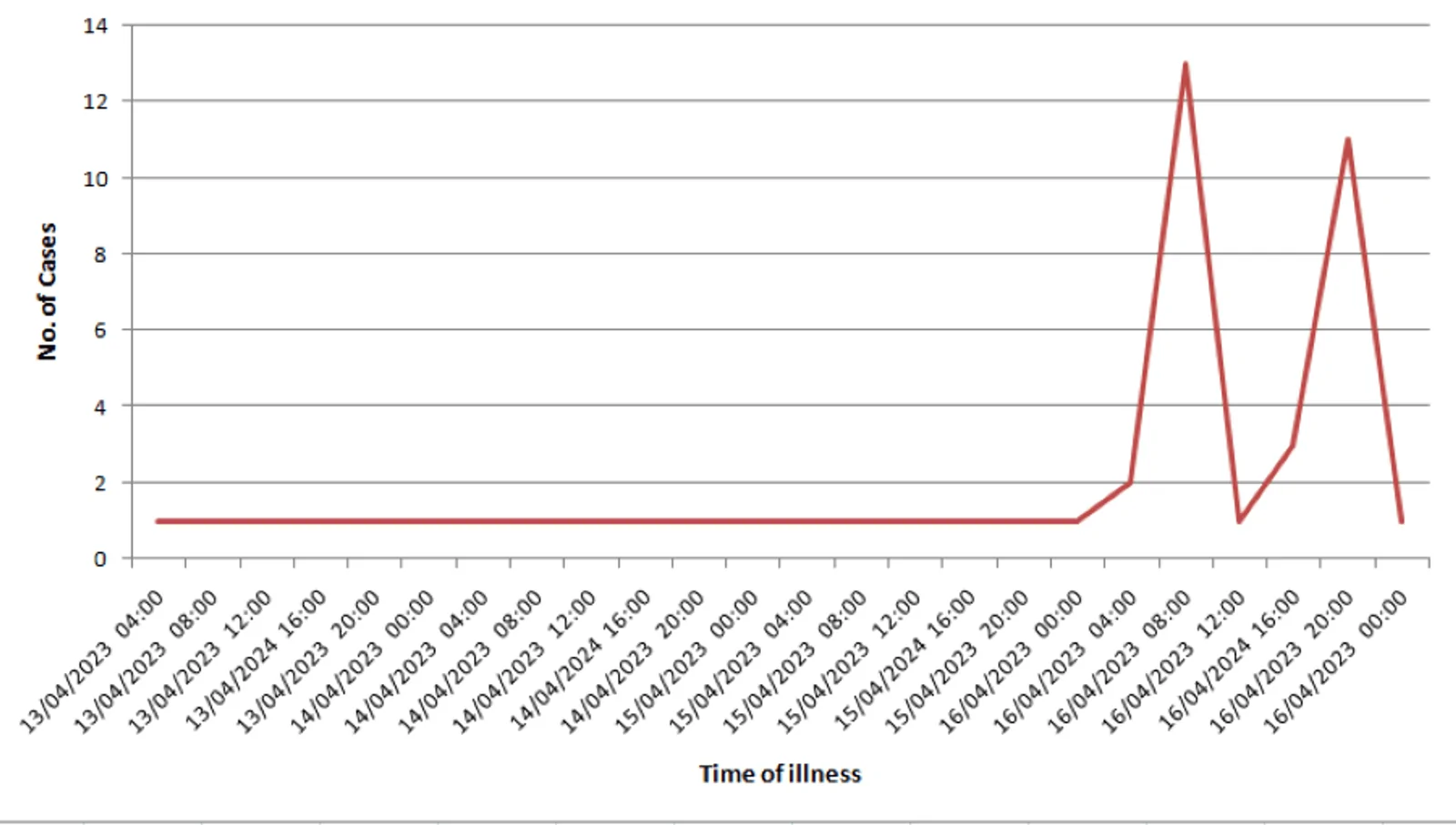
Distribution of cases with time of onset of illness The figure was created using Epi InfoTM 7, developed by the Centers for Disease Control and Prevention (CDC).

The test group comprised people who had visited the religious ceremony on 13 April, took a meal at the teashop and suffered from the disease, while the control group comprised people who had attended the religious ceremony, took a meal at the teashop, but did not suffer from the disease.

The distribution of food consumption between the two groups was compared using the chi-square test. Measures of association are expressed as odds ratios (OR) and relative risks (RR) with 95% confidence intervals (CI).

Consumption of 'Prasad' was significantly higher in the test group (98.46%) compared with that in the control group (17.14%) (χ² = 71.64, df = 1, p < 0.0001). The odds of consuming 'Prasad' were markedly higher in the test group (OR = 309.33; 95% CI: 36.69-2607.85). The relative risk indicated that participants in the test group were 5.75 times more likely to consume 'Prasad' than controls (RR = 5.75; 95% CI: 2.80-11.82). Khichri consumption was significantly higher in the control group (94.29%) compared with the test group (67.69%) (χ² = 9.09, df = 1, p = 0.003). The test group demonstrated lower odds of Khichri consumption (OR = 0.13; 95% CI: 0.03-0.61) and reduced relative risk (RR = 0.72; 95% CI: 0.59-0.89). Similarly, water consumption was significantly higher in the control group (85.71%) than in the test group (63.08%) (χ² = 5.66, df = 1, p = 0.017). The odds ratio (OR = 0.29; 95% CI: 0.10-0.89) and relative risk (RR = 0.74; 95% CI: 0.60-0.91) indicated significantly lower consumption in the test group. 'Prasad' (offering comprising chickpea or bengal gram, moong or green gram, fruits, etc., and served raw), khichri (made of rice, lentils, vegetables and ghee) and water were served in the Puja ceremony. The raw materials used to prepare the 'Prasad' were washed with water from the tubewell and soaked in tubewell water overnight before serving the next morning during the religious ceremony or 'Puja'.

The OR for ‘Prasad’, ‘Khichri’ and water were calculated to be 309.33, 0.126 and 0.284, respectively. Thus, ‘Prasad’ was considered to be the implicating food item because almost all the affected people were found to have eaten ‘Prasad’ and showed a significant OR (Table 4).

**Table 4:** Frequency of food items consumed in the ceremony (a total of 100 people), and the association of food consumption between the control group (35) and test group (65) studied using the chi-square test 'Prasad' is an offering comprising chickpea or bengal gram, moong or green gram, fruits, etc., and served raw, and 'Khichri' is a cooked dish made of rice, lentils, vegetables and ghee. CI: confidence interval; OR: odds ratio; RR: relative risk

Food item	Test group (65) n (%)	Control group (35) n (%)	χ² (df = 1)	p-value	OR (95% CI)	RR (95% CI)
Prasad	64 (98.46)	6 (17.14)	71.64	<0.0001	309.33 (36.69-2607.85)	5.75 (2.80-11.82)
Khichri	44 (67.69)	33 (94.29)	9.09	0.003	0.13 (0.03-0.61)	0.72 (0.59-0.89)
Water	41 (63.08)	30 (85.71)	5.66	0.017	0.29 (0.10-0.89)	0.74 (0.60-0.91)

Laboratory findings

Non-lactose fermenting colonies were seen growing on MacConkey agar, and black colonies appeared in the XLD agar (Figure 3) in three out of the seven stool samples after overnight incubation. Biochemical tests identified the organism as belonging to the *Salmonella *group, probably *Salmonella *Typhimurium.

**Figure 3:**
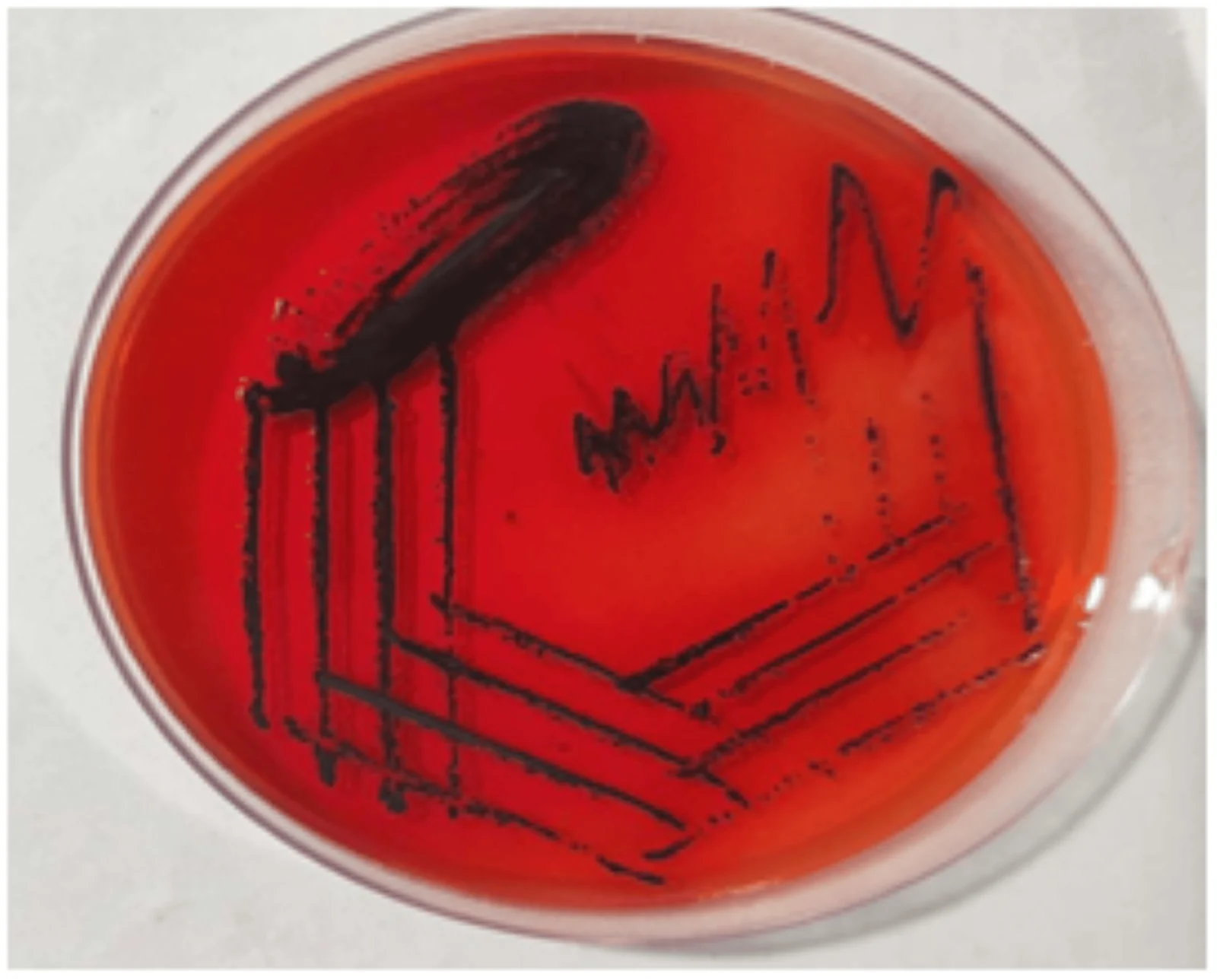
Colonies of Salmonella Typhimurium in xylose lysine deoxycholate agar

Serotyping was done with* Salmonella* antisera, and it was confirmed that the organism was *S. *Typhimurium (Figure 4). Isolation of *S. *Typhimurium (an important bacterium causing gastroenteritis) from three out of the seven stool samples implicated this organism as the causative organism of the outbreak. The isolates were sent to ICMR-NIRBI, Kolkata, where the causative agent was confirmed to be *S. *Typhimurium through serotyping and molecular techniques and their linkage to a common source. The most common symptoms reported were abdominal cramps (92.31%), fever (90.77%), followed by diarrhoea (84.62%).

**Figure 4:**
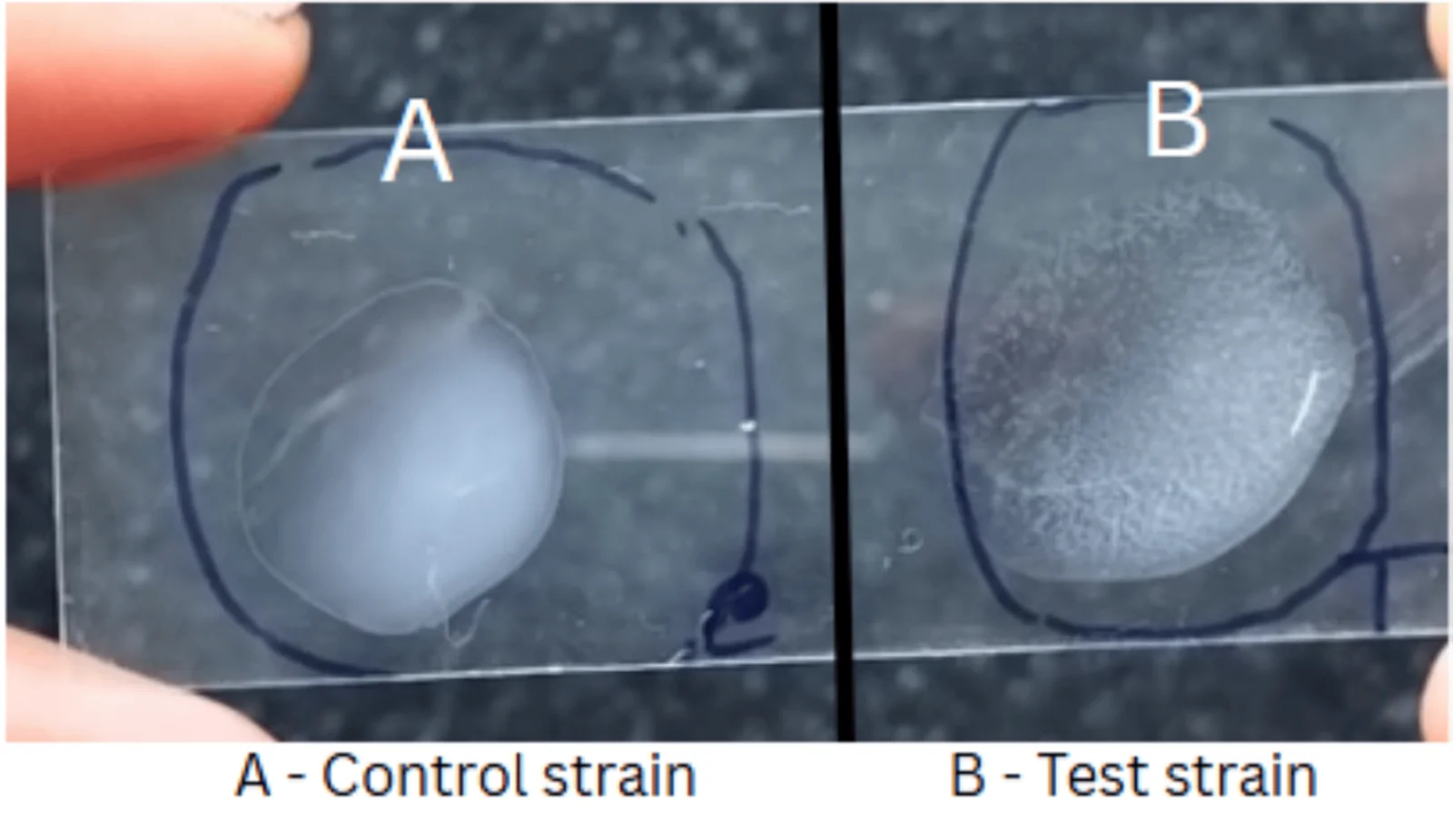
Antisera O4 positive for Salmonella Typhimurium. (A) Control strain and (B) test strain

Water analysis of both water samples was found to be unsatisfactory. Samples were processed using the multiple tube test for presumptive coliform count (or most probable number (MPN)), which indicates the presence of coliform bacteria in drinking water and potential contamination. Water sample collected from the tube-well showed the growth of 35 MPN/100 mL, while the overhead SINTEX reservoir showed growth of 17 MPN/100 mL [28]. The tube-well water (found to be unsatisfactory) was used to wash and soak the 'Prasad' items (chickpea or bengal gram, moong or green gram). 

The antibiotic sensitivity test results of the isolates are shown in Table 5.

**Table 5:** Antibiotic sensitivity report (Interpretation according to SOP) [24] SOP: standard operating procedures

Antibiotic (in µg)	Isolate 1 (*Salmonella *Typhimurium 1)		Isolate 2 (*Salmonella *Typhimurium 2)		Isolate 3 (*Salmonella *Typhimurium 3)	
	Zone size (mm)	Interpretation	Zone size (mm)	Interpretation	Zone size (mm)	Interpretation
Gentamicin (10)	23	Sensitive	22	Sensitive	23	Sensitive
Chloramphenicol (30)	16	Intermediate	17	Intermediate	16	Intermediate
Imipenem (10)	25	Sensitive	25	Sensitive	25	Sensitive
Meropenem (10)	30	Sensitive	30	Sensitive	28	Sensitive
Cefotaxime (30)	29	Sensitive	29	Sensitive	31	Sensitive
Cefepime (30)	27	Sensitive	28	Sensitive	28	Sensitive
Ceftriaxone (30)	26	Sensitive	27	Sensitive	27	Sensitive
Azithromycin (15)	18	Sensitive	18	Sensitive	19	Sensitive
Ampicillin (10)	14	Resistant	15	Resistant	14	Resistant
Ciprofloxacin (5)	28	Intermediate	27	Intermediate	28	Intermediate
Trimethoprim-sulphamethoxazole (1.25/23.75)	8	Resistant	8	Resistant	9	Resistant
Tetracycline (30)	13	Intermediate	13	Intermediate	13	Intermediate

Management of cases

The affected cases were admitted to the local health facility, i.e. Nagrijuli Model Hospital. Patients were treated with oral rehydration solution (ORS). In more severe cases, the antibiotic ceftriaxone was administered, while azithromycin was administered based on antibiotic sensitivity reports. Intravenous fluids (IVF) were also administered as needed. All patients fully recovered following the treatment.

Outcome of the outbreak

Out of a total of 65 affected people, 60 had been hospitalised. The higher proportion of hospitalisation among the symptomatic patients was done as a precautionary measure by the local health authorities to avoid any adverse outcome. But most patients were discharged the next day of admission after observation for 24 hours. All 65 patients (both admitted and non-admitted) recovered after getting fluids and antibiotics. All patients were discharged by 20 April, 2023.

## Discussion

In this current outbreak of food poisoning, food served on 13 April 2023 at the Puja ceremony at a teashop in Nagrijuli was contaminated. The epidemic curve showed a point-source outbreak, where the onset of symptoms occurred on 15 April, while most cases were reported around 4 to 8 a.m. and again between 5 to 8 p.m. on 16 April. 'Prasad' was thought to be the implicating food item, as 93% of the affected people were found to have eaten prasad. The most clinching evidence that *S.* Typhimurium is the causative agent of this outbreak is its isolation in three out of the seven stool samples collected from patients.

The clinical and epidemiological characteristics of an outbreak can help indicate its etiology. Diarrhoea, with or without abdominal cramps, is a common presenting symptom of food poisoning caused by *Salmonella* spp., *Campylobacter jejuni*, *Clostridium perfringens* and the diarrhoeal form of *Bacillus cereus*. But fever, seen in 90.77% of cases in the present outbreak, is characteristically seen only in *Salmonella* food poisoning. This pattern was also seen in the outbreak of *Salmonella* food poisoning reported by Kunwar et al. [29]. A similar outbreak caused by contamination of mayonnaise with *S.* Typhimurium was reported by Mitchell et al. in the United Kingdom, where the most common symptoms were diarrhoea, abdominal cramps and fever [30]. An outbreak of *S.* Typhimurium food poisoning having a similar pattern of illness and outcome after consumption of tofu from a particular manufacturer was reported from Canada by Osasah et al. [31]. Another* S.* Typhimurium outbreak was reported by Lehti et al. after the consumption of contaminated vegetable mix that contained iceberg lettuce, cucumber and peas served during lunch in a day care centre in Finland. The most common symptoms reported were diarrhoea (94.8%), abdominal pain (88.8%) and fever (83.6%), followed by headache (47.4%), nausea (44.8%) and vomiting (38.8%) [32].

Statistically significant differences in food consumption patterns between the test and control groups were demonstrated in our study. Consumption of 'Prasad' was markedly higher in the test group than in the control group. The strength of association was substantial, as reflected by a high OR of 309.33 and an RR of 5.75, with CIs excluding unity, indicating strong statistical significance. This suggests a robust association between group allocation and 'Prasad' consumption. Conversely, 'Khichri' consumption was significantly higher in the control group. An OR of 0.13 and an RR of 0.72 indicate that participants in the test group were significantly less likely to consume khichri. Similarly, water consumption was lower in the test group than in controls, with statistically significant measures of association. The wide CI observed for 'Prasad' (particularly for the OR) may be attributed to the very small number of non-consumers in the test group and low consumption in the control group, which can inflate the OR and reduce precision. Despite this, the RR provided a more stable and interpretable estimate in this context.

A review from Hong Kong on 195 outbreaks of food poisoning by non-typhoidal *Salmonella* species reported that the outbreaks were mainly linked to a single contaminated food item, along with inadequate cooking identified as a factor in about half of outbreaks. Along with the presence of contaminated raw ingredients, these findings suggest that, in most outbreaks of non-typhoidal *Salmonella *food poisoning, the raw food itself was the primary source of the organism [33]. This corroborates with the findings in our outbreak report, where ‘Prasad’ (raw food item) is thought to be the contributing food item. The index case developed symptoms on 15 April, that is, within 36 hours of exposure, followed by a sharp increase in the number of cases over the next 24 hours. The outbreak subsided by the next day, with the last case being reported on the morning of 17 April. The steep rise and fall of the epidemic curve indicates a point source outbreak, consistent with the patterns described by Kunwar et al. from India [29].

*Salmonella* Typhimurium isolated in the present outbreak was sensitive to gentamicin, imipenem, meropenem, cefotaxime, cefepime, ceftriaxone and azithromycin but was resistant to ampicillin and cotrimoxazole and showed intermediate sensitivity towards ciprofloxacin, chloramphenicol and tetracycline, thus showing a great degree of drug resistance overall. In an outbreak investigation by Saikia et al., *S.* Weltevreden was found to cause food poisoning in a tea garden in Assam, India, and the isolates were found to be sensitive to ampicillin, amoxicillin-clavulanic acid, ciprofloxacin, ofloxacin, norfloxacin, cefotaxime, ceftriaxone, co-trimoxazole and doxycycline [13]. In a study by Yoon et al. conducted in South Korea, *S. enteritidis* and *S. typhimurium* isolates showed the highest overall resistance (98.3%) and multidrug resistance (73.3%) amongst *Salmonella *isolates. The most commonly observed antibiotic resistance pattern among *Salmonella* spp. was towards ampicillin and chloramphenicol [34]. In another study from Iran by Kanaan, nalidixic acid resistance was the most common (73.7%), followed by resistance to sulfamethoxazole-trimethoprim (63.2%) and tetracycline (63.2%), while gentamicin and ciprofloxacin (up to 15.8%) showed comparatively lower resistance rates [35]. A meta-analysis of* S.* Typhimurium isolates to different antibiotic agents by Qin et al. in China reported resistance rate varying from very high to low as follows: tetracycline, 93%; ampicillin, 91%; nalidixic acid, 73%; trimethoprim-sulfamethoxazole, 48%; amoxicillin-clavulanic acid, 70%; chloramphenicol, 54%; gentamicin, 44%; ciprofloxacin, 22%; ceftriaxone, 21%; cefotaxime, 19%; cefepime, 12%; ceftazidime, 10% [12]. Thus, multidrug resistance among non-typhoidal *Salmonella* species, particularly *S. *Typhimurium, is being increasingly reported, which has also been found in our present outbreak isolates and is a matter of concern.

In our outbreak investigation, an environmental survey of the place where food and 'Prasad' were prepared showed that the place was very unhygienic, with poor handling and storage of the prepared food. The place was also infested with flies, cockroaches and rats, which might act as vectors for contaminating the food and spreading infection in the environment, thus emphasising the need for brisk surveillance and mass awareness regarding hygienic practices, including safe handling and storage of food.

Limitation of the outbreak investigation

The source of food contamination could not be determined due to the unavailability of food samples. Another limitation of the study is the retrospective case recall of study participants.

Recommendations

Disinfection of Drinking Water Sources

Ensuring regular and effective disinfection of all drinking water sources is essential to prevent waterborne diseases. Appropriate chlorination and routine monitoring of water quality should be implemented.

Implementation of Jal Jeevan Mission

Strengthening and accelerating the implementation of the Jal Jeevan Mission is crucial to equitable access to safe and clean drinking water for the population.

Mass Awareness on Sanitation and Hygiene

The Social Welfare and Health Departments should conduct sustained mass awareness campaigns focusing on personal hygiene, safe sanitation practices and disease prevention strategies.

Monitoring of Food Establishments

Food safety officers should carry out regular inspections and strict supervision of restaurants, tea stalls and other food establishments to ensure adherence to hygienic practices, proper food handling and effective waste disposal systems.

Active Surveillance of Diarrhoeal Diseases

Health workers should undertake continuous active surveillance of diarrhoeal cases to enable early detection, timely intervention and implementation of preventive measures to control outbreaks.

## Conclusions

To our knowledge, this investigation represents the first documented outbreak of food poisoning caused by *S.* Typhimurium in Assam. The findings confirm that this highly transmissible foodborne pathogen poses a significant public health threat, particularly in the context of increasing antimicrobial resistance. Rapid identification of the outbreak source, combined with timely dissemination of evidence-based findings to local health authorities, enabled effective control of this outbreak and improved case management. The study highlights the urgent need for targeted preventive measures, including strict adherence to food safety practices, proper hand hygiene, routine disinfection of drinking water sources and improved environmental sanitation. Strengthening these interventions will be critical to reducing the risk of similar outbreaks in the future. Furthermore, this outbreak investigation underscores the importance of rapid response teams and robust pathogen surveillance systems, such as the IDSP, particularly in under-reported regions of North-East India.

## Figures and Tables

**Figure 5:**
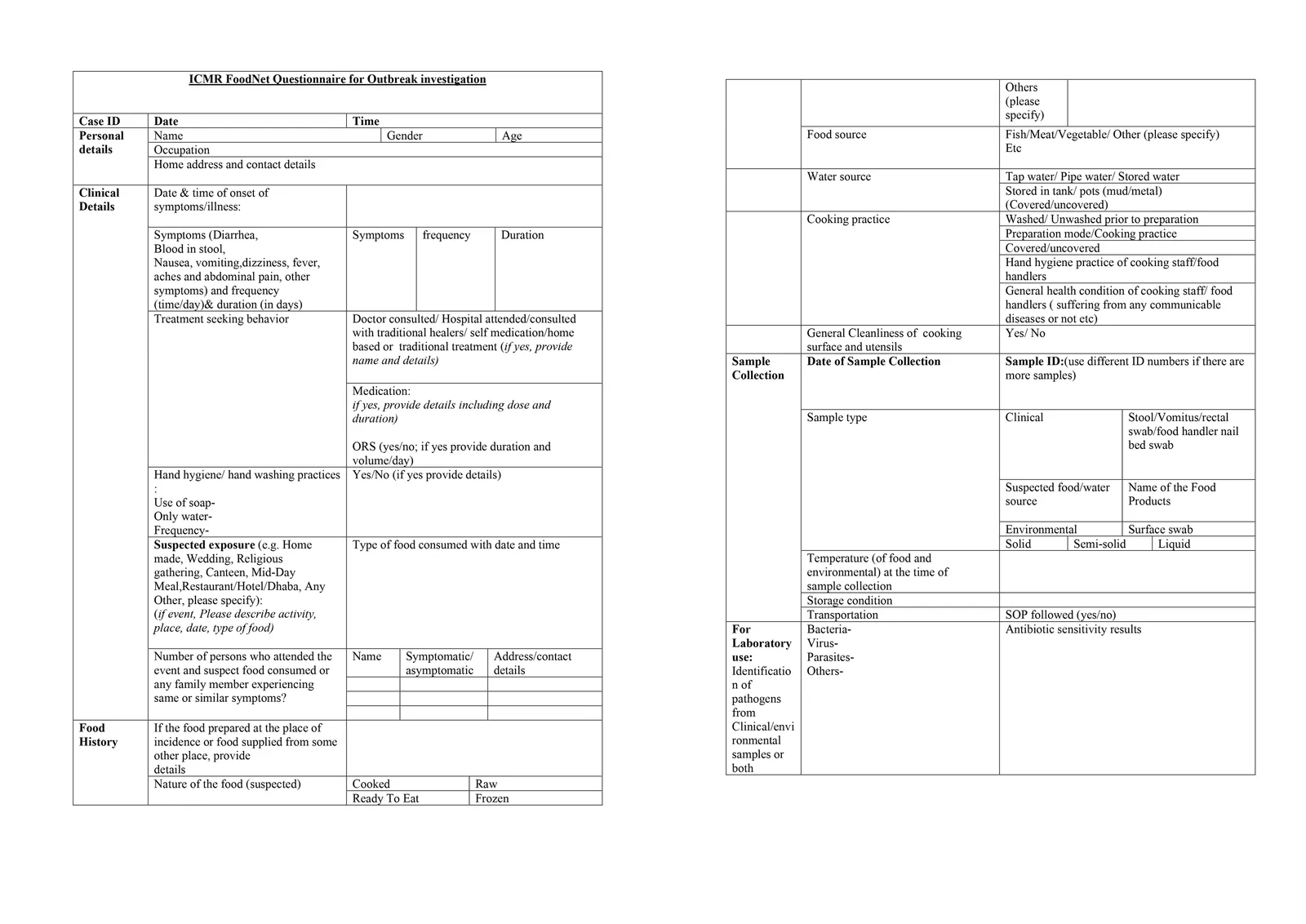
Structured questionnaire (epidemiological case sheet) used to collect demographic data
